# Prevalence and Associated Factors of Uncontrolled Hypertension Among Patients Attending Primary Healthcare Centers in Riyadh, Saudi Arabia

**DOI:** 10.7759/cureus.64783

**Published:** 2024-07-17

**Authors:** Mohammed Alshuhri, Bander Alshehry, Turki Alotaibi, Doaa Alhalal

**Affiliations:** 1 Radiology and Medical Imaging, Prince Sattam Bin Abdulaziz University, Al-Kharj, SAU; 2 Family Medicine, King Saud Medical City, Riyadh, SAU

**Keywords:** dyslipidemia, diabetes, cardiovascular diseases, poor blood pressure control, uncontrolled hypertension

## Abstract

Background

Hypertension (HTN) is a global health issue as it causes significant mortality and morbidity among the worldwide population. Various treatments are available, but many patients are unable to control the disease. There are various factors like medication non-adherence and lifestyle habits that contribute to this problem. There is a need for evidence-based interventions to address HTN effectively, especially in regions like Saudi Arabia, where there is limited data on uncontrolled HTN. This study aimed to assess the prevalence of uncontrolled HTN and contributing factors to poor blood pressure control among patients in Primary Health Centers (PHCs) in Riyadh, Saudi Arabia.

Methodology

An analytical cross-sectional study was conducted using an interviewer-administered questionnaire among all patients aged 18 years and above who have uncontrolled HTN and who visited the PHCs of Riyadh's first health cluster in Saudi Arabia. Data was cleaned in Microsoft Excel and analyzed using IBM SPSS 29.

Results

The study comprised 516 patients with HTN. The majority were males (53.1%, n=274) compared to females (46.9%, n=242), with an average age of 58 years (SD=10.5). Notably, most patients were obese (63.2%, n=326), and 62.4% (n=322) had uncontrolled HTN. Multivariate analysis identified sociodemographic factors like higher education (p-value = 0.013, adjusted odds ratio (AOR) = 0.795) as protective against uncontrolled HTN, while employment (p-value = 0.031, AOR = 1.786) increased the risk of uncontrolled HTN. Risk factors such as smoking (p-value = 0.001, AOR = 3.011) and salt restriction (p-value = 0.021, AOR = 0.643) significantly influenced HTN control. Management-related predictors like stopping medication after feeling better (p-value = 0.001, AOR = 3.196) were also found significant.

Conclusion

This study revealed a high prevalence of uncontrolled HTN, especially among males and obese individuals. Sociodemographic factors like higher education were protective, while employment increased the risk of the disease. Further, smoking, salt restriction, and medication adherence significantly impacted HTN control, highlighting the importance of tailored interventions.

## Introduction

Hypertension (HTN) is a significant public health issue and the leading cause of death worldwide. In many countries around the globe, HTN is becoming more prevalent. HTN, or increased blood pressure (BP), is a chronic disease characterized by a persistent elevation of arterial BP above normal levels [1]. According to the American College of Cardiology (ACC), the optimum systolic blood pressure (SBP) value is less than 120 mmHg, and diastolic blood pressure (DBP) is less than 80 mmHg [2]. Worldwide, 26% of the population has HTN, and the prevalence is expected to reach 29% by 2025 [3]. HTN is the most important modifiable risk factor for cardiovascular and kidney diseases and a leading cause of mortality [1, 2]. The WHO mentioned that "Worldwide, raised BP is estimated to cause 7.5 million deaths, about 12.8% of the total of all deaths” [4].

Controlling HTN at the optimum BP levels is essential for reducing cardiovascular diseases. According to the ACC and American Heart Association (AHA), a general treatment target for HTN is to maintain 130/80 mmHg of BP. Uncontrolled HTN is defined as above or equal to 140/90 mmHg; (160/90 mmHg for patients over 60 years of age). Despite treatment with at least two optimal antihypertensive agents at maximum doses and adequate treatment time [5], and the availability of effective treatment, only 21% of adults with HTN had it under control [6]. Lack of knowledge and poor self-care habits make HTN more challenging to manage and increase its impact.

In Saudi Arabia, according to the most recent survey, the prevalence of HTN among the population was 31.4% [7]. According to the Global Burden of Disease 2010 study, untreated and uncontrolled HTN is a primary risk factor for ischemic heart disease and mortality in Saudi Arabia [8].

Uncontrolled HTN remains a significant public health and economic burden in both developed and developing countries due to premature deaths and cardiovascular disabilities. Previous studies identified numerous factors that influence poor BP control, including non-adherence to medication, failure of lifestyle modification, smoking, alcohol intake, and comorbidities such as diabetes, obesity, and kidney diseases. Additionally, poor lifestyle behaviors and socioeconomic characteristics were shown to predict poorly controlled BP [9-12].

Implementing evidence-based, adherence-enhancing, and healthy lifestyle programs is essential; therefore, a proper understanding of the nature of the disease and analyzing the impact of patient behaviors are needed to establish health strategies for effective HTN prevention and control. Unfortunately, there is limited data about uncontrolled HTN and its associated factors in Saudi Arabia. Therefore, this study aimed to estimate the prevalence of uncontrolled HTN and to determine the contributing factors of poor BP control among adults in primary care centers (PHCs) of the first health cluster in Riyadh, Saudi Arabia, thereby establishing health strategies for effective HTN prevention and control.

## Materials and methods

Study design and study setting

The present analytical cross-sectional research was conducted using an interviewer-administered questionnaire to determine the vulnerability of patients with uncontrolled hypertension who visited the PHCs of Riyadh's first health cluster in Saudi Arabia from February 2024 to May 2024.

Eligibility criteria

A diversified randomized procedure was used to select the overall number of hypertension patients from the medical records of individuals registered at each clinic of the PHCs in Riyadh, Saudi Arabia. Therefore, adults above the age of 18 years who were diagnosed with hypertension and were on pharmacological therapy for hypertension were included in the study. However, participants under the age of 18 years and those who were not receiving any pharmacological therapy for controlling hypertension were excluded.

Sampling technique

Patients were recruited using random sampling techniques.

Sample size calculation

The study included individuals who met the specified inclusion and exclusion criteria. The sample size was determined using the Roasoft online sample size calculator, tailored for epidemiological analysis, factoring in a 95% CI level, a 5% margin of error, and the total population based on the registered hypertensive patients in the PHCs of Riyadh (n=2000). The sample size was found to be 323, but data was collected from 516 individuals to accommodate the non-response rate.

Data collection tool and method

A newly created interviewer-administered questionnaire was used for data collection. Before its use, a pilot study was conducted with a sample of 25 participants, whose results were not included in the final analysis. Necessary adjustments were made to the questionnaire to ensure the questions were clear and understandable. The questionnaire was adjusted to better fit the research needs. After conducting the pilot study, questions were added to identify other comorbidities associated with HTN, such as diabetes mellitus, dyslipidemia, and smoking.

The survey was completed in person during face-to-face meetings. Oral informed consent was obtained before data collection. Clinical information of all enrolled patients was retrieved from the hospital's electronic Raqeem system, including baseline demographic data (gender, BMI, age), and vital signs measurements (including systolic pressure and diastolic pressure) at two clinic visits.

Measurable variables

The measurable variables include the mean systolic pressure (mmHg), mean diastolic pressure (mmHg), and BMI (kg/m²). Blood pressure measurements were conducted in the vitals room following the patients' appointment registration. Each patient was given a rest period of 10-15 minutes prior to the measurement to ensure accuracy. The diagnosis of HTN was made in accordance with the guidelines established by the AHA. Uncontrolled HTN was defined as SBP ≥ 140 mmHg or DBP ≥ 90 mmHg.

Additionally, the BMI of each patient was classified based on the following ranges (kg/m²): less than 18.5 as underweight, 18.5 to 24.9 as normal weight, 25 to 29.9 as overweight, 30 to 34.9 as obese class 1, 35 to 39.9 as obese class 2, and 40 or more as obese class 3. This classification provided a comprehensive understanding of the patients' weight status in relation to their HTN diagnosis.

Data analysis plan

A comprehensive statistical analysis was conducted on the dataset, encompassing both descriptive and inferential methodologies. Firstly, a descriptive analysis was performed to summarize the demographic characteristics of the participants, including age, gender, and other features, providing an overview of the study population. Subsequently, inferential analyses, such as the multivariate binary logistic regression model, were employed to identify the adjusted predictors of uncontrolled HTN. Statistical significance was established at a p-value of 0.05 or lower and a 95% CI. All statistical analyses were executed using IBM's SPSS Software, version 29.0.0.

Ethical consideration

The Institutional Review Board of King Saud Medical City approved the research via reference number H0RI-08-Feb24-04, dated 15/02/2024. Data was collected and registered by the person assigned by the Principal Investigator (PI). The data was anonymized and protected. Investigators guaranteed that clinical data would be used for clinical research purposes only.

## Results

This study included 516 patients who were assessed for uncontrolled HTN and its risk factors. There was a higher proportion of males (n=274, 53.1%) compared to females (n=242, 46.9%). The average age was 58 years (SD=10.5), ranging from 18 to 93 years. Patients' average height was 162.2 cm (SD=11.0), ranging from 75 to 184 cm, and average weight was 86.7 kg (SD=18.8), ranging from 50 to 155 kg. A mean BMI of 32.8 kg/m2 (SD=7.0) was found, ranging from 20.4 to 70 kg/m2. Most patients were classified as obese (n=326, 63.2%), while 27.5% (n=142) were overweight. The average SBP was 140.9 mmHg (SD=17.8), ranging from 100 to 197 mmHg; and the DBP was 79.5 mmHg (SD=12.3), ranging from 53 to 127 mmHg. The majority of patients were Saudi nationals (n=444, 86.0%). Regarding education, 41.1% (n=212) had a high school education or above. Employment status showed that 76.0% (n=196) were unemployed (Table 1).

**Table 1:** Sociodemographic and other parameters of patients (n=516) The data is presented as frequency (n) and percentages (%).

Sociodemographic variables		Frequency (Percentage) N (%)
Gender	Female	242 (46.9)
	Male	274 (53.1)
Nationality	Non-Saudi	72 (14.0)
	Saudi	444 (86.0)
Education	No Formal Education	128 (24.8)
	Primary	104 (20.2)
	Elementary	72 (14.0)
	≥ High School	212 (41.1)
Occupation	Unemployed	392 (76.0)
	Employed	124 (24.0)
BMI Category	Underweight/Normal	48 (9.3)
	Overweight	142 (27.5)
	Obese	326 (63.2)

**Figure 1:**
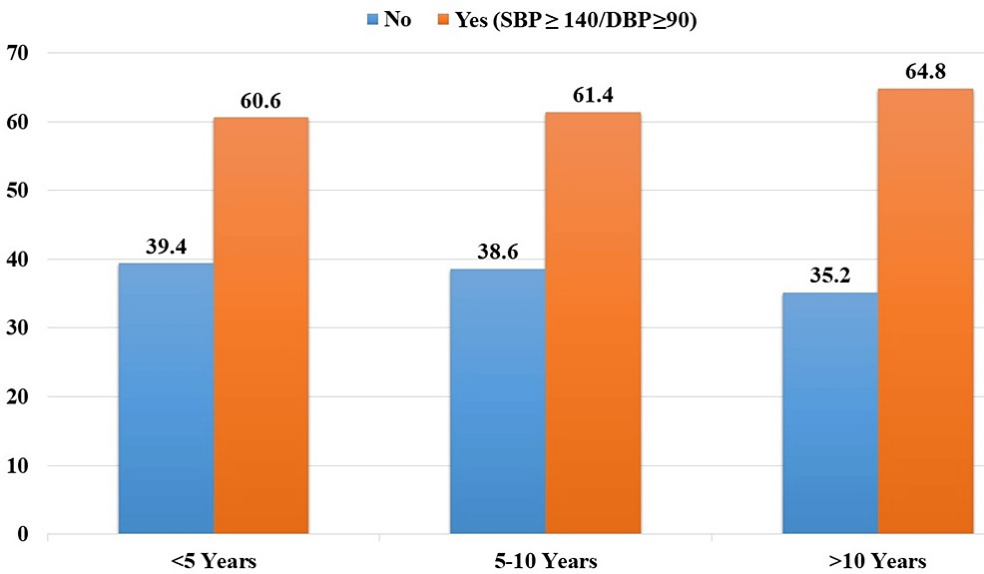
Uncontrolled hypertension by duration (n=516). SBP: Systolic blood pressure.

Among patients with HTN for less than 5 years, 60.6% (n=120) had uncontrolled HTN. For those with HTN lasting 5 to 10 years, 61.4% (n=86) had uncontrolled HTN. In patients with HTN for more than 10 years, the prevalence of uncontrolled HTN was highest at 64.8% (n=114) (Figure 1).

It was found that 62.4% (n=322) of the patients had uncontrolled HTN, defined as SBP ≥ 140/DBP ≥ 90. Regarding medication adherence, 41.5% (n=214) reported forgetting to take their medications. Additionally, 20.5% (n=106) stopped their medication when feeling better. A majority of patients (56.6%, n=292) were on monotherapy. On average, patients engaged in 30 minutes of exercise for 2.1 days (SD=2.4) in the past week, with specific exercises like swimming or walking for an average of 1.9 days (SD=2.4) during the past seven days (Table 2).

**Table 2:** Prevalence of uncontrolled hypertension, medications, and exercise-related features (n=516). The data is presented as frequency (n) and percentages (%). SBP: Systolic blood pressure; DBP: Diastolic blood pressure.

		Frequency (Percentage) N (%)
Uncontrolled Hypertension (SBP ≥ 140/DBP ≥90)	No	194 (37.6)
	Yes	322 (62.4)
Do you ever forget to take your medications	No	302 (58.5)
	Yes	214 (41.5)
Do you ever have problems remembering to take your medication	No	358 (69.4)
	Yes	158 (30.6)
When you feel better you stop your medication	No	410 (79.5)
	Yes	106 (20.5)
If you feel worse when you take your medication do you stop taking it	No	444 (86.0)
	Yes	70 (13.6)
Number of drugs	Mono	292 (56.6)
	Combination	224 (43.4)

**Figure 2:**
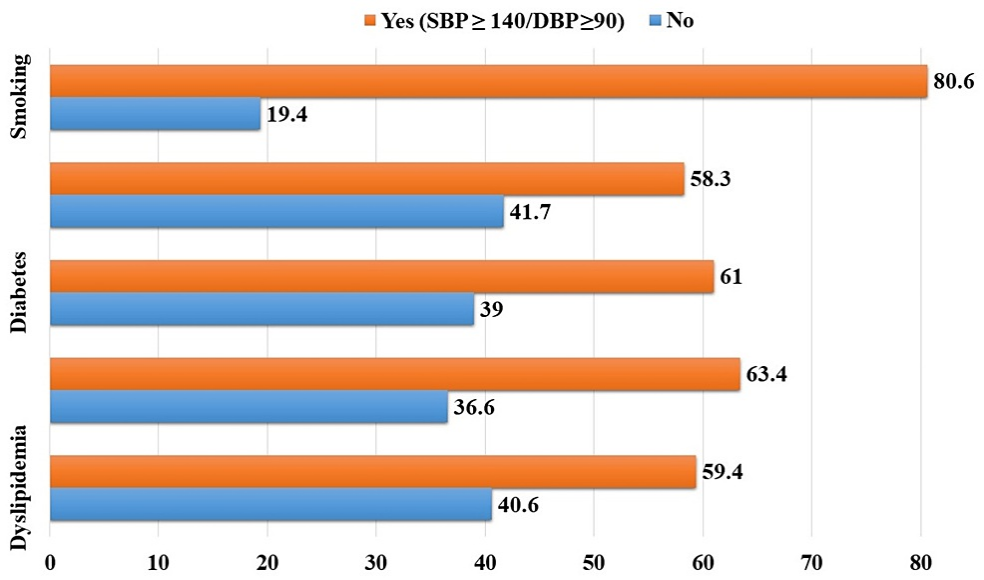
Risk factors of HTN among participants (n=516). SBP: Systolic blood pressure; DBP: Diastolic blood pressure; HTN: Hypertension.

Among those with dyslipidemia, 59.4% (n=222) had HTN (SBP ≥ 140/DBP ≥ 90). Participants with a family history of HTN exhibited a similar trend, with 63.4% (n=232) having HTN. For individuals with diabetes, 61% (n=200) had HTN. Lack of salt restriction was associated with HTN in 58.3% (n=176) of cases. Notably, smoking appeared to be strongly correlated with HTN, as 80.6% (n=50) of smokers had HTN (Figure 2).

All variables were coded and treated as continuous variables; the lowest value (0) was used as a reference value, for example, female, non-Saudi, and unemployed participants were the reference groups in each relevant category. Age was not found to be a significant predictor of having uncontrolled HTN (B = 0.006, p-value = 0.599, adjusted odds ratio (AOR) = 1.006, 95% CI: 0.985-1.027). Males had a higher likelihood of having uncontrolled HTN (B = 0.456, p-value = 0.072, AOR = 1.578, 95% CI: 0.959-2.596), although this was not statistically significant. Saudi nationality also did not significantly predict uncontrolled HTN (B = 0.212, p-value = 0.492, AOR = 1.237, 95% CI: 0.675-2.267). Higher education was found to be a protective factor, significantly associated with lower odds of uncontrolled HTN (B = -0.229, p-value = 0.013, AOR = 0.795, 95% CI: 0.664-0.952). Employment status significantly increased the odds of uncontrolled HTN (B = 0.580, p-value = 0.031, AOR = 1.786, 95% CI: 1.055-3.023). Similarly, BMI (overweight) was not significantly associated with uncontrolled HTN (B = 0.053, p-value = 0.103, AOR = 1.054, 95% CI: 0.989-1.123) (Table 3).

**Table 3:** Adjusted sociodemographic predictors of uncontrolled HTN (Logistic multivariate regression analysis). *Considered significant at p-value less than 0.05. HTN: Hypertension.

	Exp (B)	P-value	Adjusted Odds Ratio (AOR)	95% Confidence Interval (CI)	
				Lower	Upper
Age	0.006	0.599	1.006	0.985	1.027
Gender (Male)	0.456	0.072	1.578	0.959	2.596
Nationality (Saudi)	0.212	0.492	1.237	0.675	2.267
Higher Education	-0.229	0.013*	0.795	0.664	0.952
Employment	0.580	0.031*	1.786	1.055	3.023
Height (cm)	0.012	0.390	1.012	0.985	1.039
Weight (kg)	-0.012	0.301	0.988	0.965	1.011
BMI (Overweight)	0.053	0.103	1.054	0.989	1.123

Except for salt restriction, where 'Yes' was set as the reference value, 'No' was set as the reference value in all groups. Smoking was strongly associated with an increased likelihood of uncontrolled HTN (B = 1.102, p-value = 0.001, AOR = 3.011, 95% CI: 1.546-5.864), indicating that smokers were three times more likely to have uncontrolled HTN. Salt restriction showed a protective effect (B = -0.442, p-value = 0.021, AOR = 0.643, 95% CI: 0.441-0.936), suggesting that patients who practiced salt restriction were less likely to have uncontrolled HTN. Family history of HTN (B = 0.164, p-value = 0.427, AOR = 1.178, 95% CI: 0.786-1.764) and diabetes (B = 0.054, p-value = 0.803, AOR = 1.055, 95% CI: 0.690-1.615) were not significant predictors. Dyslipidemia showed a trend towards significance, indicating a potential protective effect, but was not statistically significant (B = -0.448, p-value = 0.062, AOR = 0.639, 95% CI: 0.399-1.022) (Table 4).

**Table 4:** Adjusted risk factors of uncontrolled HTN (logistic multivariate regression analysis). *Considered significant at p-value less than 0.05. HTN: Hypertension.

	Exp (B)	P-value	Adjusted Odds Ratio (AOR)	95% Confidence Interval (CI)	
				Lower	Upper
Smoking	1.102	0.001*	3.011	1.546	5.864
Salt Restriction	-0.442	0.021*	0.643	0.441	0.936
Family History of HTN	0.164	0.427	1.178	0.786	1.764
Diabetes	0.054	0.803	1.055	0.690	1.615
Dyslipidemia	-0.448	0.062	0.639	0.399	1.022

For all categorical variables, 'No' was set as a reference value. Forgetting to take medication was not a significant predictor (B = 0.048, p-value = 0.819, AOR = 1.049, 95% CI: 0.695-1.583). Similarly, having problems remembering to take medication did not significantly affect HTN control (B = -0.333, p-value = 0.133, AOR = 0.717, 95% CI: 0.465-1.107). However, stopping medication after feeling better was a significant predictor (B = 1.162, p-value = 0.001, AOR = 3.196, 95% CI: 1.644-6.210), indicating that patients who stopped medication when feeling better were over three times more likely to have uncontrolled HTN. Again, stopping medication after feeling worse was not a significant predictor (B = -0.449, p-value = 0.235, AOR = 0.638, 95% CI: 0.304-1.339). The days with specific exercises like swimming or walking showed a trend towards significance (B = -0.250, p-value = 0.061, AOR = 0.779, 95% CI: 0.599-1.011), suggesting a potential protective effect, but it was not statistically significant (Table 5).

**Table 5:** Adjusted management-related predictors of uncontrolled HTN (logistic multivariate regression analysis). *Considered significant at p-value less than 0.05. HTN: Hypertension.

	Exp (B)	P-value	Adjusted Odds Ratio (AOR)	95% Confidence Interval (CI)	
				Lower	Upper
Forget to take medicine	0.048	0.819	1.049	0.695	1.583
Problem in remembering medicine	-0.333	0.133	0.717	0.465	1.107
Stop medication after feeling better	1.162	0.001*	3.196	1.644	6.210
Stop medication after feeling worse	-0.449	0.235	0.638	0.304	1.339
No. of drugs (Poly)	0.249	0.199	1.283	0.877	1.877
No. of days with 30 minutes exercise during past 7 days	0.200	0.134	1.221	0.941	1.585
No. of days with 30 specific exercises (swimming/walking) during the past 7 days	-0.250	0.061	0.779	0.599	1.011

## Discussion

Notably, this study showed that there is a very high prevalence of uncontrolled HTN, with 62.4% of patients exhibiting uncontrolled blood pressure. Abdisa L et al. (2022) revealed a high magnitude of uncontrolled HTN at 48% [13]. This finding is consistent with previous research indicating suboptimal HTN control rates, particularly in primary care settings. Teh XR et al. (2020) indicated that BP control was suboptimal and deficient in the process of care with consequent gaps between guidelines and actual clinical practices [14]. Notably, there was a slightly higher proportion of males in the current study sample, although gender did not emerge as a significant predictor of uncontrolled HTN in the multivariate analysis but showed a trend of higher likelihood of uncontrolled HTN among males. This contrasts with some studies that reported a higher prevalence of HTN among males. Santosa A et al. (2020) showed that Swedish and Chinese men had lower control of HTN than women [15]. Gillis EE and Sullivan JC (2016) showed that although both men and women develop HTN, distinct gender differences in the incidence and severity of HTN are well established, with men having a higher incidence and poorer control of HTN compared with women of the same age until the sixth decade of life [16]. This highlighted the need for further investigation into gender-specific factors influencing HTN control.

Regarding age as a predictor of uncontrolled HTN, the current study findings did not show any association of HTN control with advancing age. However, previous literature indicates that control of HTN becomes poor with advancing age. An article published by the National Institute of Aging showed that high blood pressure, or HTN, is a major health problem that is common in older adults. The older the age of an individual, the stiffer the arteries, causing blood pressure to increase [17].

Furthermore, this study did not show an association between nationality and uncontrolled HTN. However, there is an established relationship between ethnicities and races with HTN prevalence and its control. Fei KeZhen FK et al. (2017) showed that black and Asian adults had significantly greater odds of HTN than whites [18].

Notably, the current study revealed that higher education was a protective factor for uncontrolled HTN. This aligns with previous literature that education plays an important role in the maintenance of the health of an individual. In the current study, the type of job was not mentioned, so we cannot describe it as a confounding factor, while age was not a significant factor contributing to the occurrence of uncontrolled HTN in the current study after adjustment for other factors. Sun K et al. (2022) showed that participants with basic education exhibited excess risks of newly diagnosed HTN and worse blood pressure control compared with individuals with higher education [19]. Abdisa L et al. (2022) revealed that patients with high blood pressure who had not completed formal education were three times more likely to have uncontrolled HTN [20].

Notably, the current study findings showed that employment was a significant predictor of uncontrolled HTN. Similarly, Lavigne-Robichaud M et al. (2019) demonstrated that workers/employees exposed to psychosocial stressors at work according to the demand-latitude model had a higher prevalence of uncontrolled HTN [21].

Notably, several clinical characteristics and comorbidities were identified as potential risk factors for uncontrolled HTN. Most notably, the majority of the population was obese, with a BMI of 32.8 (63.2%), illustrating obesity as a factor for HTN and its impact on HTN control. Praso S et al. (2012) showed that persistent obesity directly raises blood pressure and makes its control more difficult by interfering with the effectiveness of antihypertensive drugs [22]. Similarly, Hammami R et al. (2021) indicated that obesity is an increasing health problem and a strong predictor of uncontrolled BP [23]. Uncontrolled HTN due to obesity might be influenced by various factors, including lifestyle behaviors, genetic predisposition, and access to healthcare. The presence of comorbid conditions such as dyslipidemia, diabetes, and a family history of HTN was common among the study patients, although they were not significant predictors of uncontrolled HTN in the multivariate analysis.

Behavior-related factors including smoking, salt restriction, and medication adherence emerged as significant predictors of uncontrolled HTN. Notably, smoking was strongly correlated with HTN, and smokers were three times more at risk of poor blood pressure control compared to non-smokers. In contrast, Zhang C et al. (2020) showed no correlations between smoking and blood pressure control in people who were diagnosed with HTN [24]. Wagai GA et al. (2023) observed a rise in blood pressure levels in the presence of high smoking rates among participants [25]. These findings highlight the importance of smoking cessation interventions in HTN management programs. Tsai SY et al. (2021) demonstrated that smoking cessation programs significantly yield a reduction in both systolic and diastolic blood pressure [26]. Moreover, salt restriction was also a critical factor in HTN control because patients who practiced salt restriction were less likely to have uncontrolled HTN. De Keyzer W et al. (2015) showed that reduced sodium intake can lower blood pressure after four weeks in unstable or uncontrolled hypertensive patients [27]. Pimenta E et al. (2009) indicated that excessive dietary sodium ingestion contributes importantly to resistance to antihypertensive treatment [28].

Medication adherence was also a critical factor in HTN control because patients who stopped taking their medication without completing the full course of treatment were three times more likely to have uncontrolled HTN. Contrarily, Pallangyo P et al. (2022) noted that there was suboptimal blood pressure control among participants despite a satisfactory adherence rate, suggesting a need for lifestyle modifications which play a central role in HTN management [29]. Thus, there is a need to develop interventions for comprehensive patient education and counseling on the importance of continued treatment adherence and lifestyle modification among hypertensive patients.

Several limitations were considered when interpreting the findings of this study. The cross-sectional design limits the ability to establish causality or temporal relationships between variables. Longitudinal studies are needed to explore the dynamic nature of HTN control and the trajectory of risk factors over time. Additionally, the current study was conducted in a specific primary care setting, which might limit the generalizability of the findings to other populations or healthcare settings. Future research could include diverse populations and settings to enhance the external validity of the findings.

## Conclusions

This study provided valuable insights into the prevalence of uncontrolled HTN and its associated risk factors among patients visiting primary care settings in Riyadh, Saudi Arabia. The findings underscore the importance of comprehensive HTN management strategies that address both clinical and behavioral factors. By targeting modifiable risk factors such as smoking and medication adherence, healthcare providers can work towards improving HTN control rates and reducing the burden of cardiovascular morbidity and mortality associated with uncontrolled HTN. Future research should focus on implementing and evaluating tailored interventions aimed at addressing the multifaceted nature of HTN control in diverse patient populations.
